# Supplementary material for: Dynamic transcriptomic profiles of zebrafish gills in response to zinc supplementation
Source: BMC Genomics. 2010 Oct 11;11:553. doi: 10.1186/1471-2164-11-553 (PMC3091702; doi:10.1186/1471-2164-11-553)
Supplement: Additional file 2 — Interactive Direct Interaction Network representing the molecular interactions between zinc, copper, iron, calcium and proteins encoded by transcripts changed by zinc supplementation. Mini web-site containing index.html and hyperlinked pages in subdirectory describing a Direct Interaction Network automatically generated based on curated interactions contained within the proprietary PathwayArchitect database. Ovals represent proteins and the circles symbolize metal ions. Objects are coloured by their abundance in zebrafish at the time-point they were significantly different from the control is a scale from -4 fold (dark green) to +4 fold (dark red). Where significant differences were found at more than one time-point, the colour overlay shows expression at the first instance. Dark blue squares denote 'binding', and light blue squares 'expression'; green squares stand for 'regulation', green diamonds for 'metabolism', and green circles for 'promoter binding'. Arrow heads indicate directionality of the interaction where annotated. All nodes and edges can be further interrogated by selecting the relative area of the image. [file 1471-2164-11-553-S2.zip › PathwayArchitect Zn xs DIN/434720.html]

# METABOLISM:

|  |  |
| --- | --- |
| Type | METABOLISM |
| Effect | None |


---

|  |  |
| --- | --- |
| Score | 0 |


---

|  |  |
| --- | --- |
| Reference Count | 5 |


---

|  |  |
| --- | --- |
| Mechanism | Unknown |


---

|  |  |
| --- | --- |
| Reference:0 || Sentence | "Since Cu and Zn are necessary for the synthesis of copper-zinc superoxide dismutase (CuZnSOD), which disables the noxious O2-, the deficiencies cause considerable damage as we age." |
| PMID | 11399105 |
| Year | 2001 |
| Species | Human |
| Journal | Med Hypotheses |
| RefScore | 2 |
| Source | PArchNLP |
  |
|  |  |


---

|  |  |
| --- | --- |
 Reference:1 || Sentence | "Zinc, by inducing intestinal metallothionein, inhibits absorption of copper from food, and inhibits reabsorption of endogenously secreted copper, thereby producing a negative copper balance in Wilson's disease." |
| PMID | 3886759 |
| Year | 1985 |
| Species | Human |
| Journal | J Am Coll Nutr |
| RefScore | 2 |
| Source | PArchNLP |
  ||  |  |


---

|  |  |
| --- | --- |
 Reference:2 || Sentence | "Transfection of a pSV2 human copper-zinc superoxide dismutase expression vector into murine fibroblasts resulted in stable transgenic clones producing increased amounts of copper-zinc superoxide dismutase." |
| PMID | 9101240 |
| Year | 1995 |
| Species | Mouse |
|  | Human |
| Journal | Free Radic Biol Med |
| RefScore | 1 |
| Source | PArchNLP |
  ||  |  |


---

|  |  |
| --- | --- |
 Reference:3 || Sentence | "The effects of moderately elevated zinc (Zn) intake on copper (Cu) metabolism and on the pancreas were studied." |
| PMID | 1384853 |
| Year | 1992 |
| Species | Mouse |
| Journal | J Trace Elem Electrolytes Health Dis |
| RefScore | 0 |
| Source | PArchNLP |
  ||  |  |


---

|  |  |
| --- | --- |
 Reference:4 || Sentence | "Effects of metallothionein (MT) synthesis inhibiting compounds (actinomycin D, cycloheximide), MT synthesis stimulating compounds (dexamethasone, dibu-cAMP) and interfering metals (Cd, Zn) on copper accumulation were investigated in rat hepatoma tissue culture cells." |
| PMID | 7940570 |
| Year | 1994 |
| Species | Rat |
| Journal | Toxicology |
| RefScore | 1 |
| Source | PArchNLP |
  |


---

|  |  |
| --- | --- |
